# *Caenorhabditis elegans* F-Box Protein Promotes Axon Regeneration by Inducing Degradation of the Mad Transcription Factor

**DOI:** 10.1523/JNEUROSCI.1024-20.2021

**Published:** 2021-03-17

**Authors:** Tatsuhiro Shimizu, Strahil I. Pastuhov, Hiroshi Hanafusa, Yoshiki Sakai, Yasuko Todoroki, Naoki Hisamoto, Kunihiro Matsumoto

**Affiliations:** Division of Biological Science, Graduate School of Science, Nagoya University, Nagoya 464-8602, Japan

**Keywords:** axon regeneration, *C. elegans*, Mad/Max, ubiquitin

## Abstract

In *Caenorhabditis elegans*, axon regeneration is activated by a signaling cascade through the receptor tyrosine kinase (RTK) SVH-2. Axonal injury induces *svh-2* gene expression by degradation of the Mad-like transcription factor MDL-1. In this study, we identify the *svh-24*/*sdz-33* gene encoding a protein containing F-box and F-box-associated domains as a regulator of axon regeneration in motor neurons. We find that *sdz-33* is required for axon injury-induced *svh-2* expression. SDZ-33 targets MDL-1 for poly-ubiquitylation and degradation. Furthermore, we demonstrate that SDZ-33 promotes axotomy-induced nuclear degradation of MDL-1, resulting in the activation of *svh-2* expression in animals. These results suggest that the F-box protein is required for RTK signaling in the control of axon regeneration.

**SIGNIFICANCE STATEMENT** In *Caenorhabditis elegans*, axon regeneration is positively regulated by the growth factor SVH-1 and its receptor tyrosine kinase SVH-2. Expression of the *svh-2* gene is induced by axonal injury via the Ets-like transcription factor ETS-4, whose transcriptional activity is inhibited by the Mad-like transcription factor MDL-1. Axon injury leads to the degradation of MDL-1, and this is linked to the activation of ETS-4 transcriptional activity. In this study, we identify the *sdz-33* gene encoding a protein containing an F-box domain as a regulator of axon regeneration. We demonstrate that MDL-1 is poly-ubiquitylated and degraded through the SDZ-33-mediated 26S proteasome pathway. These results reveal that an F-box protein promotes axon regeneration by degrading the Mad transcription factor.

## Abstract (precis)

In *Caenorhabditis elegans*, axon regeneration is activated by a signaling cascade through the receptor tyrosine kinase (RTK) SVH-2. Axonal injury induces *svh-2* gene expression by degradation of the Mad-like transcription factor MDL-1. In this study, we identify the *svh-24*/*sdz-33* gene encoding a protein containing F-box and F-box-associated domains as a regulator of axon regeneration in motor neurons. We find that *sdz-33* is required for axon injury-induced *svh-2* expression.

## Introduction

The ability of axons to regenerate after damage is a fundamental and conserved property of neurons, modulated by intrinsic processes that regulate axon growth potential. Upon axon severance, regenerative signals are transmitted from the sites of damage to the nucleus, whereupon several transcription factors are upregulated along with the synthesis of proteins participating in neurite outgrowth (Tedeschi and Bradke, 2017). Since manipulation of these signaling processes can improve the likelihood of successful axon regeneration, these processes are potential targets for regenerative therapies. However, these intrinsic signaling mechanisms have yet to be fully elucidated.

The nematode *Caenorhabditis elegans* has recently emerged as an attractive model to dissect the mechanisms of axon regeneration in the mature nervous system (Yanik et al., 2004). In *C. elegans*, the JNK MAP kinase (MAPK) cascade plays a crucial role in the initiation of axon regeneration (Nix et al., 2011; Hisamoto and Matsumoto, 2017). This pathway consists of MLK-1 MAPKKK, MEK-1 MAPKK, and KGB-1 JNK, and is inactivated at the KGB-1 activation step by VHP-1, a member of the MAPK phosphatase family (Mizuno et al., 2004). The *vhp-1*-null mutation causes hyperactivation of the JNK pathway, resulting in developmental arrest at an early larval stage. We recently have identified new components functioning in JNK-mediated signaling by using a genome-wide RNAi screen for suppressors of *vhp-1* lethality (*svh* genes; Li et al., 2012). The *svh-1* gene encodes a growth factor-like protein homologous to mammalian hepatocyte growth factor (HGF), and the *svh-2* gene encodes a homolog of mammalian Met, a receptor for HGF (Li et al., 2012). SVH-2 is a receptor tyrosine kinase (RTK) that activates the JNK pathway via the tyrosine phosphorylation of MLK-1 MAPKKK.

**Figure 1.**
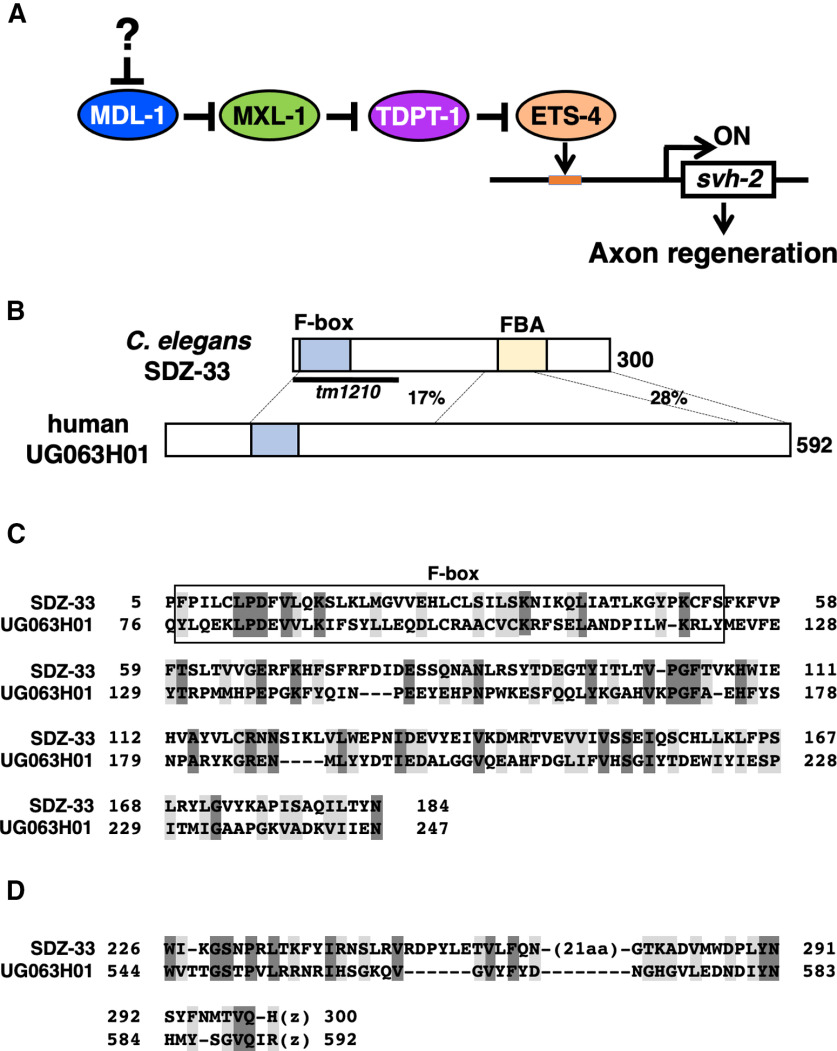
Identification of SDZ-33. ***A***, Regulation of *svh-2* expression in response to axon injury. MXL-1 forms a complex with MDL-1, and TDPT-1 interacts with ETS-4 to induce its SUMOylation, resulting in the repression of ETS-4 transcriptional activity. Axon injury leads to the degradation of MDL-1, allowing free MXL-1 to interact with TDPT-1. ETS-4 is then de-SUMOylated, and subsequently induces *svh-2* expression. ***B***, Structures of SDZ-33 and human UG063H01. The domains shown are the F-box domain (blue) and type 2 FBA (yellow). Percentages of similarities are indicated. The bold line underneath indicates the extent of the deleted region in the *tm1210* deletion mutant. ***C***, ***D***, Amino acid alignments of the N-terminal (***C***) and C-terminal (***D***) domains. Identical and similar residues are highlighted with dark and pale gray shading, respectively.

SVH-1−SVH-2 signaling specifically regulates axon regeneration, and this specificity is determined by the induction of *svh-2* gene expression following axon injury (Li et al., 2012). This upregulation critically involves the physical interaction of the Ets-like transcription factor ETS-4 and the CCAAT/enhancer-binding protein (C/EBP)-like transcription factor CEBP-1 (Li et al., 2015). Upon axon injury, cAMP levels increase in severed neurons, resulting in the activation of cAMP-dependent protein kinase (PKA), which in turn phosphorylates ETS-4. Phosphorylated ETS-4 is able to form a complex with CEBP-1, which then activates *svh-2* transcription (Li et al., 2015). Furthermore, we recently identified the Mad-like transcription factor MDL-1, the Max-like transcription factor MXL-1, and TDP2 (tyrosyl-DNA phosphodiesterase 2)-like TDPT-1 as components involved in the regulation of ETS-4 transcriptional activity for the induction of *svh-2* gene expression (Fig. 1*A*; Sakai et al., 2019). TDPT-1 interacts with and induces SUMOylation of ETS-4, which interferes with PKA-mediated phosphorylation of ETS-4. As a result, formation of the ETS-4–CEBP-1 complex is inhibited, and its transcriptional activity thereby repressed. MXL-1 activates *svh-2* transcription by interacting with TDPT-1 and relieving inhibition of ETS-4 activity. MDL-1 forms a complex with MXL-1, and this interaction induces the dissociation of TDPT-1 from MXL-1, enabling free TDPT-1 to inhibit ETS-4 transcriptional activity. Thus, TDPT-1 and MDL-1 negatively regulate axonal injury-induced expression of the *svh-2* gene via modulation of ETS-4 (Fig. 1*A*). Axon injury leads to the degradation of MDL-1, which is linked to the activation of ETS-4 transcriptional activity. Thus, MDL-1 protein stability is important in the regulation of axon regeneration, but the details of how this stability is modulated are at present unknown.

In the present study, we investigate the role of the *svh-24*/*sdz-33* gene in the regulation of axon regeneration. The *sdz-33* gene encodes a protein containing an F-box domain (Robertson et al., 2004), which confers substrate recognition by the SCF [S-phase kinase-associated protein 1 (Skp1)–Cullin1–F-box] E3-ubiquitin (Ub) ligases (Craig and Tyers, 1999). Here, we show that MDL-1 is recognized by SDZ-33, which directs its degradation via the 26S proteasome. Thus, induction of the SDZ-33-mediated MDL-1 degradation pathway following neuron injury is essential for axon regeneration.

## Materials and Methods

### 

#### 

##### *C. elegans* strains.

The *C. elegans* strains used in this study are listed in Table 1. All strains were maintained on nematode growth medium plates and fed with bacteria of the OP50 strain, as described previously (Brenner, 1974).

**Table 1.** Strains used in this study

Strain	Genotype
KU501	*juIs76 II*
KU503	*juIs76 II; svh-2(tm737) X*
KU1533	*juIs76 II; sdz-33(tm1210) III*
KU1534	*juIs76 II; sdz-33(tm1210) III; kmEx1534 [Psdz-33::sdz-33::sdz-33 3'UTR]*
KU1535	*juIs76 II; sdz-33(tm1210) III; kmEx1535 [Punc-25::sdz-33::sdz-33 3'UTR]*
KU1536	*juIs76 II; sdz-33(tm1210) III; kmEx1536 [Punc-25::svh-2]*
KU1537	*juIs76 II; sdz-33(tm1210) III; svh-2(tm737) X*
KU1538	*tdpt-1(km68) I; juIs76 II; sdz-33(tm1210) III*
KU1539	*juIs76 II; sdz-33(tm1210) III; mdl-1(tm311) X*
KU1527	*kmEx1527 [Punc-25::cfp* + *Psvh-2::nls::venus]*
KU1540	*sdz-33(tm1210) III; kmEx1527 [Punc-25::cfp* + *Psvh-2::nls::venus]*
KU1541	*sdz-33(tm1210) III; mdl-1(tm311) X; kmEx1527 [Punc-25::cfp* + *Psvh-2::nls::venus]*
KU1542	*mdl-1(tm311) X; kmEx1527 [Punc-25::cfp* + *Psvh-2::nls::venus]*
KU1543	*wpIs36 I; kmEx1543 [Punc-25::mdl-1::gfp]*
KU1544	*wpIs36 I; sdz-33(tm1210) III; kmEx1543 [Punc-25::mdl-1::gfp]*
KU1545	*wpIs36 I; kmEx1545 [Psdz-33::nls::gfp]*
KU1546	*juIs76 II; sdz-33(tm1210) III; kmEx1546 [Punc-25::sdz-33(cDNA)::unc-54 3'UTR]*
KU1547	*juIs76 II; sdz-33(tm1210) III; kmEx1547 [Pmec-7::sdz-33::sdz-33 3'UTR]*

##### Plasmids.

The *Psdz-33::sdz-33::sdz-33 3′UTR* clone was generated by PCR amplification of ∼1.7 kb of the *sdz-33* gene from genomic DNA (using the primers 5′-tgcaaattagccaagaaacagagattgttc-3′ and 5′-cgctcaccgtatttcctgtgc-3′) and inserted into the TOPO vector (Thermo Fisher Scientific). *Psdz-33::nls::gfp* was constructed by Gibson assembly of the PCR-amplified *Psdz-33* promoter sequence (using the above-described *Psdz-33::sdz-33* sequence as a template) and the *nls::gfp*-containing vector pPD95.67. The *Punc-25::sdz-33::sdz-33* 3′*UTR* (3′ untranslated region) plasmid was constructed by replacing the *sdz-33* promoter of the *Psdz-33::sdz-33::sdz-33 3′UTR* with the *unc-25* promoter, which was amplified from the pSC325 vector. The *Punc-25::sdz-33* (*cDNA*) plasmid was constructed by inserting a DNA sequence corresponding to the *sdz-33* cDNA (synthesized by Eurofins) into the pSC325 vector. The *Pmec-7::sdz-33::sdz-33 3′UTR* plasmid was generated by PCR amplification of the *sdz-33* sequence from the *Punc-25::sdz-33::sdz-33 3′UTR* construct (using the primers 5′-atagctagcatggctactgtcccttttcctattc-3′ and 5′-tttggtacccgctcaccgtatttcctgtgc-3′), digestion with NheI/KpnI and ligation with NheI/KpnI-digested *Pmec-7* vector pPD52.102. To construct Flag-SDZ-33, the *sdz-33* cDNA was subcloned into the pCMV-Flag vector. Myc-MDL-1 was constructed by inserting the *mdl-1* cDNA into the pCMV-Myc-N vector (Clontech). *Punc-25::svh-2*, *Punc-25::cfp*, *Psvh-2::nls::venus*, *Punc-25::mdl-1::gfp*, *Pmyo-2::dsred-monomer*, and HA-Ub plasmids were described previously (Hanafusa et al., 2011; Li et al., 2012, 2015; Sakai et al., 2019).

##### Transgenic animals.

Transgenic animals were obtained by the standard *C. elegans* microinjection method (Mello et al., 1991). *Psdz-33::sdz-33::sdz-33 3′UTR*, *Psdz-33::nls::gfp*, *Punc-25::sdz-33::sdz-33 3′UTR*, *Punc-25::sdz-33* (*cDNA*)*::unc-54 3′UTR*, *Pmec-7::sdz-33::sdz-33 3′UTR*, *Punc-25::svh-2*, *Punc-25::mdl-1::gfp*, and *Pmyo-2::dsred-monomer* plasmids were used in *kmEx1534* [*Psdz-33::sdz-33::sdz-33 3′UTR* (25 ng) + *Pmyo-2::dsred-monomer* (25 ng)], *kmEx1545* [*Psdz-33::nls::gfp* (25 ng) + *Pmyo-2::dsred-monomer* (25 ng)], *kmEx1535* [*Punc-25::sdz-33::sdz-33 3′UTR* (12.5 ng) + *Pmyo-2::dsred-monomer* (5 ng)], *kmEx1546* [*Punc-25::sdz-33* (*cDNA*)*::unc-54 3′UTR* (12.5 ng) + *Pmyo-2::dsred-monomer* (5 ng)], *kmEx1547* [*Pmec-7::sdz-33::sdz-33 3′UTR* (12.5 ng) + *Pmyo-2::dsred-monomer* (5 ng)], *kmEx1536* [*Punc-25::svh-2* (25 ng) + *Pmyo-2::dsred-monomer* (5 ng)], and *kmEx1543* [*Punc-25::mdl-1::gfp* (25 ng) + *Pmyo-2::dsred-monomer* (5 ng)]. The *kmEx1527* [*Punc-25::cfp* + *Psvh-2::nls::venus* + *Pmyo-2::dsred-monomer*] extrachromosomal array and *wpIs36* integrated array were described previously (Firnhaber and Hammarlund, 2013; Sakai et al., 2019).

##### Microscopy.

Standard fluorescent images of transgenic animals were obtained under a 100× objective on a Nikon ECLIPSE E800 fluorescent microscope and photographed with a Zyla CCD camera. Confocal fluorescent images were taken on a Zeiss LSM-800 confocal laser-scanning microscope with a 63× objective.

##### Axotomy.

Axotomy and microscopy were performed as described previously (Pastuhov et al., 2012). All animals were subjected to axotomy at the young adult stage. Imaged commissures that had growth cones or small branches present on the proximal fragment were counted as “regenerated.” Proximal fragments that showed no change after 24 h were counted as “no regeneration.” A minimum of 20 individuals with one to three axotomized commissures were observed for most experiments.

##### Measuring the length of regenerating axons.

The length of regenerating axons for D-type motor neurons was measured using the segmented line tool of ImageJ. Measurements were made from the site of injury to the tip of the longest branch of the regenerating axon. Axons that did not regenerate were excluded from the measurements.

##### Biochemical experiments using mammalian cells.

Transfected HEK293 or COS-7 cells were incubated with or without MG132 (10 μm; Sigma-Aldrich) for 4.5 h. Cells were lysed in RIPA buffer [50 mm Tris–HCl, pH 7.4, 0.15 m NaCl, 0.25% deoxycholic acid, 1% NP-40, 1 mm EDTA, 1 mm dithiothreitol, phosphatase inhibitor cocktail 2 (Sigma-Aldrich), and protease inhibitor cocktail (Sigma-Aldrich)], followed by centrifugation at 15,000 × *g* for 12 min. The supernatant was added to 50 µl (1.5 mg) of Dynabeads Protein G (Thermo Fisher Scientific) with the indicated antibodies (each antibody was used at 5 µg/sample) and rotated for 2 h at 4°C. The beads were then washed three times with ice-cold PBS and subjected to immunoblotting. Antibodies and their suppliers were as follows: anti-Flag (catalog #M2; Sigma-Aldrich), anti-Myc (catalog #9E10 or #A14; Santa Cruz Biotechnology), and anti-HA (catalog #56; MBL).

##### Quantification of VENUS expression.

Expression of VENUS fluorescence was quantified using the ImageJ program (NIH). Cell bodies of severed or unsevered D neurons were outlined with closed polygons, and the mean fluorescent intensities of VENUS and CFP were measured to obtain *I*_VENUS_ and *I*_CFP,_ respectively. As a control, the cell body next to the cell body of interest was similarly analyzed [*I*_VENUS(c)_ and *I*_CFP(c)_]. To determine the background intensity of each cell, the same polygon was placed in the area neighboring the cell body and fluorescence was measured [*I*_VENUS(BG)_, *I*_VENUS(c)(BG)_, *I*_CFP(BG)_, and *I*_CFP(c)(BG)_, respectively]. The ratio of background-subtracted VENUS to CFP intensity was calculated as [*I*_VENUS_ – *I*_VENUS(BG)_]/[*I*_CFP_ – *I*_CFP(BG)_] and [*I*_VENUS(c)_ – I_VENUS(c)(BG)_]/[*I*_CFP(c)_ – *I*_CFP(c)(BG)_], respectively. The normalized relative intensity (*I*_r_) was calculated as {[*I*_VENUS_ – *I*_VENUS(BG)_]/[*I*_CFP_ – *I*_CFP(BG)_]}/{[*I*_VENUS(c)_ – *I*_VENUS(c)(BG)_]/[*I*_CFP(c)_ – *I*_CFP(c)(BG)_]}. Data were plotted using the R function box plot.

##### Time-lapse imaging of MDL-1::GFP after axotomy.

Animals expressing mCherry and MDL-1::GFP in their D-type motor neurons were imaged for 6 h at 15 min intervals beginning shortly after axotomy of selected motor neuron axons.

##### Quantitative measures of fluorescence intensity for MDL-1 degradation.

Animals expressing mCherry and MDL-1::GFP in their D-type motor neurons were imaged shortly after (0 h) and 6 h after axotomy of selected motor neuron axons. An LSM800 (Zeiss) confocal microscope was used to obtain *z*-stacks of fluorescent images for mCherry and MDL-1::GFP. The mean intensities of MDL-1::GFP and mCherry in the nuclei of neurons with severed axons were measured by drawing a circular ROI in the middle of the cell and using the measure function of ImageJ. Background intensities were measured near the measured cells. Relative MDL-1::GFP intensity (RI_MDL-1_) was calculated by dividing the background-subtracted value for GFP by the corresponding background-corrected value for mCherry at 6 h postaxotomy divided by the corresponding value at 0 h postaxotomy. The RI_MDL-1_ values for the wild-type and *sdz-33* mutant were plotted and checked for significant differences (Wilcoxon rank-sum exact test) using RStudio.

##### Statistical analysis.

Statistical analyses were conducted as described previously (Pastuhov et al., 2012). Briefly, confidence intervals (95%) were calculated using the modified Wald method, and two-tailed *p* values were calculated using Fisher's exact test (http://www.graphpad.com/quickcalcs/contingency1/). Welch's *t* test and the Wilcoxon rank-sum test (two-tailed) were performed using a *t* test calculator (https://www.graphpad.com/quickcalcs/ttest1/) and the R function wilcox.test, respectively.

##### Homology search, phylogenetic analysis, identification of domains, and alignment of amino acids.

A homology search, identification of conserved domains, and alignment of amino acids were performed using the National Center for Biotechnology Information (NCBI) DELTA-BLAST, NCBI CD-search, and GENETYX-MAC programs, respectively.

## Results

### SVH-24/SDZ-33 is required for efficient axon regeneration

To identify additional components functioning in the JNK pathway regulation of axon regeneration, we previously undertook a genome-wide RNAi screen for suppressors of *vhp-1* lethality and isolated 92 *svh* genes (Table 2). To identify components involved in axonal injury-induced degradation of MDL-1, we asked whether any of the *svh* genes encode factors that participate in protein degradation. Among these *svh* genes, we focused on *svh-24*, which encodes a protein containing an F-box domain.

**Table 2.** List of *svh* genes

*svh* gene	Name	Mammal homolog	Homolog type	Reference
*svh-1*		HGF/plasminogen	Growth factor	Hisamoto et al., 2014
*svh-2*		c-Met	RTK	Li et al., 2012
*svh-3*	*faah-1*	FAAH	Lipid amide hydrolase	Pastuhov et al., 2012
*svh-4*	*ddr-2*	DDR	RTK	Hisamoto et al., 2016
*svh-5*		Ets	Transcription factor	Li et al., 2015
*svh-6*	*tns-1*	Tensin	Scaffolding protein	Hisamoto et al., 2019
*svh-8*	*cebp-1*	C/EBP	Transcription factor	Li et al., 2015
*svh-9*	*nstp-1*	SLC35B4	Sugar transporter	Shimizu et al., 2019
*svh-10*	*sqv-3*	B4GALT7	Sugar transferase	Shimizu et al., 2019
*svh-11*		FUT	Sugar transferase	Shimizu et al., 2019
*svh-12*	*egl-30*	Gqα	heterotrimeric G protein	Pastuhov et al., 2012
*svh-13*	*ttr-11*		Lipid-binding protein	Hisamoto et al., 2018
*svh-14*	*mxl-1*	Max	Transcription factor	Sakai et al., 2019
*svh-15*	*brc-2*	BRCA2	Oncogene	Shimizu et al., 2018
*svh-19*	*let-502*	ROCK	Protein kinase	Shimizu et al., 2018
*svh-20*	*emb-9*	COL4A5	Collagen	Hisamoto et al., 2016

**Figure 2.**
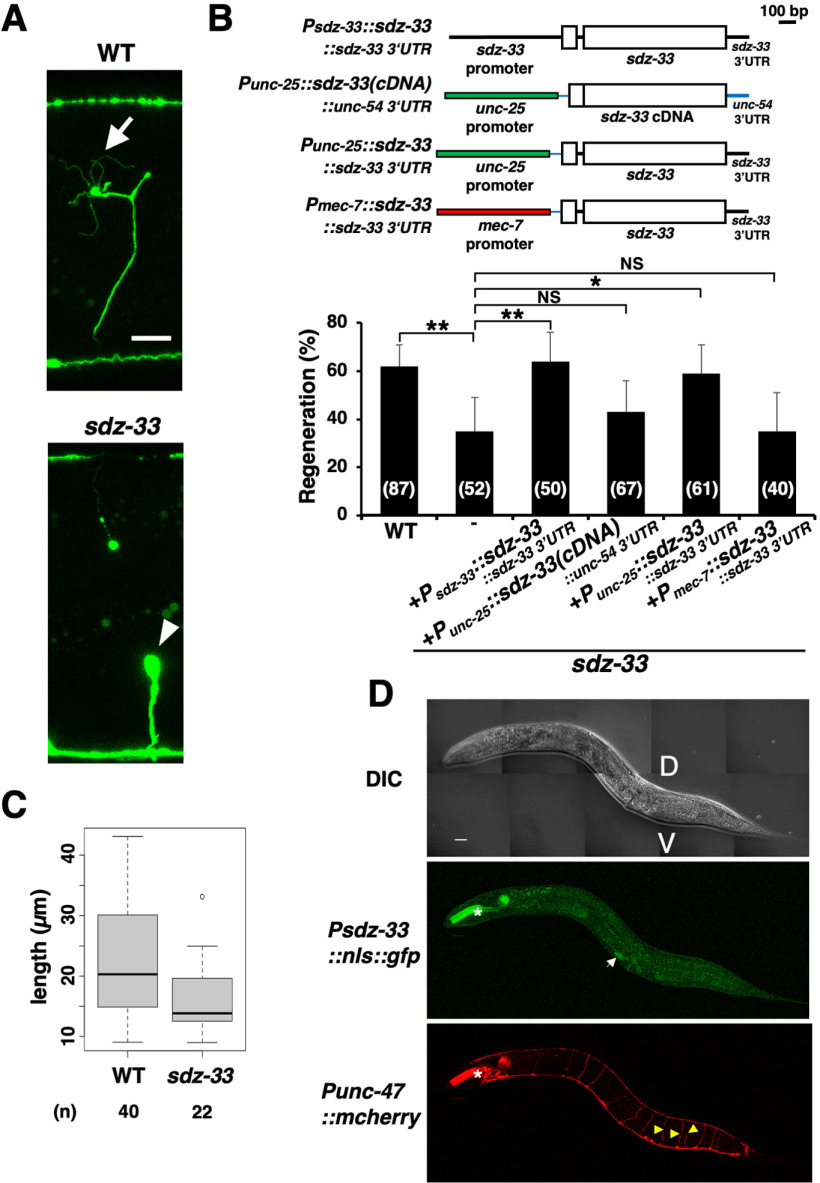
SDZ-33 is involved in axon regeneration. ***A***, Representative D-type motor neurons in wild-type (WT) and *sdz-33* mutant animals 24 h after laser surgery. In wild-type animals, a severed axon has regenerated a growth cone (arrow). In mutants, the proximal end of axon failed to regenerate (arrowhead). Scale bar, 10 µm. ***B***, Percentages of axons that initiated regeneration 24 h after laser surgery. The numbers (*n*) of axons examined are shown. Error bars indicate 95% confidence intervals. *p < 0.05, **p < 0.01, as determined by Fisher's exact test. NS, Not significant. Schematic diagrams for *Psdz-33::sdz-33::sdz-33 3′UTR*, *Punc-25:: sdz-33* (c*DNA*)*::unc-54 3′UTR*, *Punc-25::sdz-33::sdz-33 3′UTR*, and *Pmec-7::sdz-33::sdz-33 3′UTR* are shown in the top part. ***C***, Length of regenerating axons 24 h after laser surgery. The data are presented as box plot graph with median (thick line within the box) and interquartile range (edge of box). A white circle indicates an outlier. The numbers (*n*) of axons examined are shown. Statistical significance was determined using the Wilcoxon rank-sum test; *p* = 0.0036. ***D***, Expression of the *Psdz-33::nls::gfp* gene. Fluorescent and differential interference contrast (DIC) images of animals carrying *Psdz-33::nls::gfp* and *Punc-47::mcherry* 6 h after cutting are shown. D-type motor neurons are visualized by mCherry under control of the *unc-47* promoter. White arrow indicates vulval muscles. Yellow arrowheads indicate severed axons. The fluorescent signals in the pharynx (asterisks) are from the injection marker *Pmyo-2::DsRed-monomer*. V, Ventral side; D, dorsal side. Scale bar, 20 µm.

The *svh-24* gene is identical to the *sdz-33* gene, which has been identified as a ubiquitously expressed early zygotic transcript (Robertson et al., 2004). The SDZ-33 protein contains F-box and type 2 F-box-associated (FBA) domains (Fig. 1*B*; Thomas, 2006), and has weak homology with the human F-box protein UG063H01 (Fig. 1*C*,*D*). The *sdz-33(tm1210)* deletion is a null allele (Fig. 1*B*). To characterize the role of *sdz-33* in axon regeneration, we performed laser axotomy and assayed regeneration in GABA-releasing D-type motor neurons. In young adult wild-type animals, 62% of the severed axons initiated regeneration within 24 h after axon injury, whereas in *sdz-33(tm1210)* mutant animals, this frequency was reduced (Fig. 2*A*,*B*, Table 3). Although the morphology of D-type motor neurons was normal in *sdz-33* mutants, the size of bulb-like structures in the nonregenerating axons of *sdz-33* mutants was larger than that in wild-type animals (Fig. 2*A*). We found that the length of regenerating axons in *sdz-33* mutants was shorter than that observed in wild-type animals (Fig. 2*C*). However, *sdz-33* mutants had no other detectable developmental or behavioral phenotypes. To verify that the *sdz-33* mutation was responsible for this defect in axon regeneration, we generated the transgene *Psdz-33::sdz-33::sdz-33* 3′*UTR*, which contains the entire genomic *sdz-33* region (Fig. 2*B*). Introduction of *Psdz-33::sdz-33::sdz-33* 3′*UTR* into *sdz-33(tm1210)* mutants rescued the defect in axon regeneration (Fig. 2*B*; Table 3).

**Table 3.** Raw data of genotypes tested by axotomy

Strain	Genotype (*juIs76* background)	Animals, *n*	Axons, *n*	Regenerations, *n* (% of total)	*P* Value	Compared with
KU501	Wild type	49	87	54 (62%)		
KU1533	*sdz-33(tm1210)*	23	52	18 (35%)	0.0027	KU501
KU1534	*sdz-33(tm1210);Ex[Psdz-33::sdz-33::sdz-33 3'UTR]*	22	50	32 (64%)	0.0053	KU1533
KU1535	*sdz-33(tm1210); Ex[Punc-25::sdz-33::sdz-33 3'UTR]*	23	61	36 (59%)	0.0138	KU1533
KU1546	*sdz-33(tm1210); Ex[Punc-25::sdz-33(cDNA)::unc-54 3'UTR]*	26	67	29 (43%)	0.3523	KU1533
KU1547	*sdz-33(tm1210); Ex[Pmec-7::sdz-33::sdz-33 3'UTR]*	14	40	14 (35%)	1	KU1533
KU1536	*sdz-33(tm1210); Ex[Punc-25::svh-2]*	21	54	35 (65%)	0.0034	KU1533
KU503	*svh-2(tm737)*	30	54	13 (24%)	0.0002	KU501
KU1537	*sdz-33(tm1210); svh-2(tm737)*	27	55	17 (31%)	0.8369	KU1533
KU1538	*tdpt-1(km68); sdz-33(tm1210)*	27	61	37 (61%)	0.0081	KU1533
KU1539	*sdz-33(tm1210); mdl-1(tm311)*	24	58	33 (57%)	0.0226	KU1533

To investigate whether SDZ-33 functions in D-type motor neurons, we examined the expression pattern of the *sdz-33* gene. We constructed a transgene, *Psdz-33::nls::gfp*, which expresses the fluorescent protein GFP fused to a nuclear localization signal (NLS) under the control of the *sdz-33* promoter. In the young adult stage, animals carrying *Psdz-33::nls::gfp* exhibited weak expression of GFP in vulval muscles but not in D-type motor neurons. GFP expression was still not observed in D neurons after axon injury (Fig. 2*D*). Therefore, we examined whether SDZ-33 acts in D-type motor neurons. In *sdz-33(tm1210)* mutants, expression of the *sdz-33* cDNA driven by the *unc-25* promoter with the *unc-54* 3′UTR could not rescue the *sdz-33 (tm1210)* defect in axon regeneration (Fig. 2*B*, Table 3). On the other hand, when *sdz-33::sdz-33 3′UTR* was expressed by the *unc-25* promoter in *sdz-33(tm1210)* mutants, the regeneration defect was rescued (Fig. 2*B*, Table 3). These results suggest that the intron or 3′UTR region of the *sdz-33* gene is required for *sdz-33* expression. It is known that axon injury promotes stability and local translation of the mRNA encoding CEBP-1, a homolog of mammalian C/EBP, via its 3′UTR (Yan et al., 2009). This suggests that the 3′UTR region of the *sdz-33* gene may determine the stability and proper localization of the *sdz-33* mRNA. In contrast to the expression of *sdz-33::sdz-33* 3′*UTR* from the *unc-25* promoter, expression of the *sdz-33::sdz-33* 3′*UTR* DNA from the *mec-7* promoter in sensory neurons failed to rescue the *sdz-33(tm1210)* defect (Fig. 2*B*, Table 3). Thus, SDZ-33 regulates axon regeneration in injured D-type motor neurons after laser axotomy in a cell-autonomous manner.

### SDZ-33 is involved in axotomy-induced *svh-2* expression in injured D-type motor neurons

**Figure 3.**
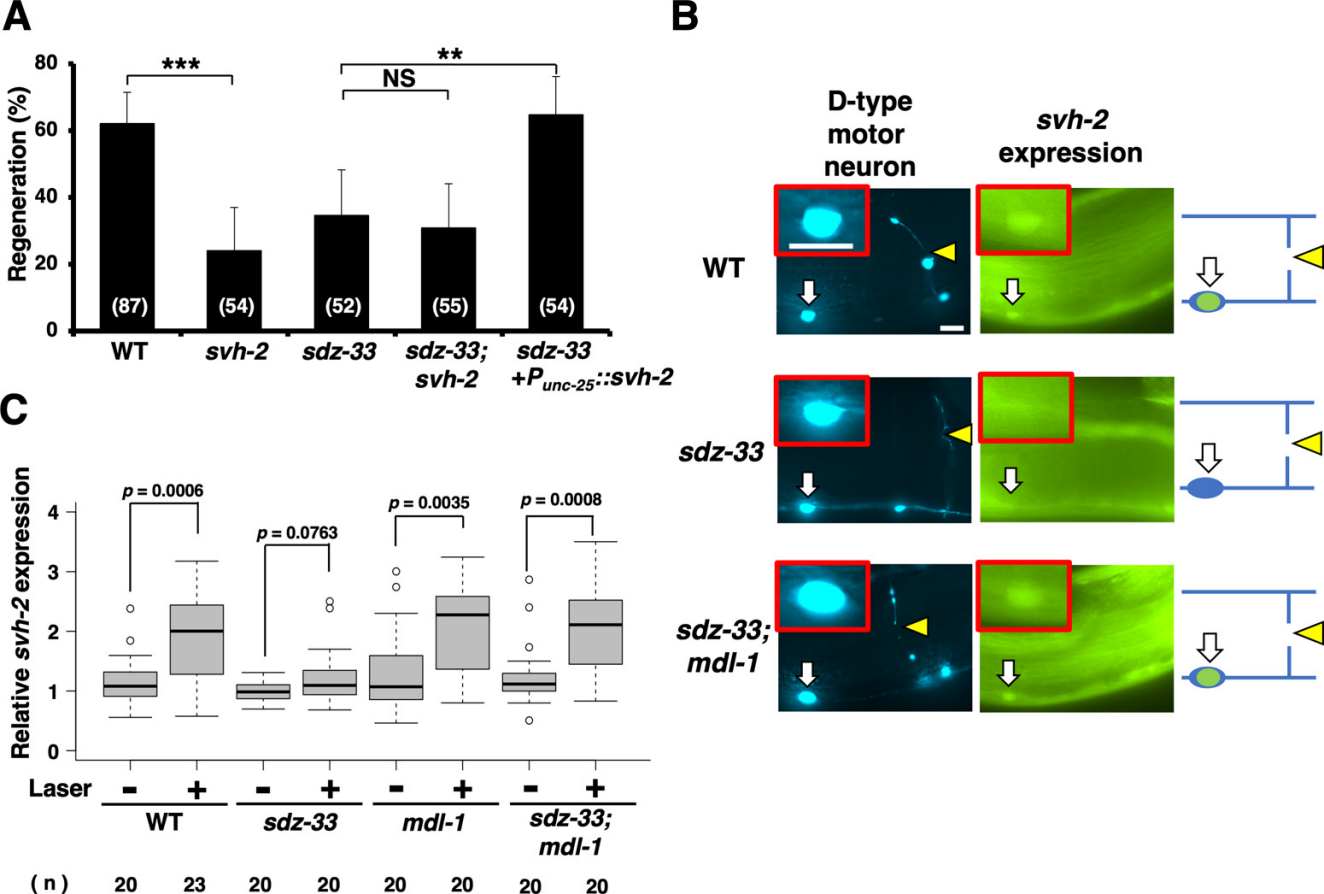
SDZ-33 is required for transcriptional induction of the *svh-2* gene in response to axon injury. ***A***, Percentages of axons that initiated regeneration 24 h after laser surgery. The numbers (*n*) of axons examined are shown. Error bars indicate 95% confidence intervals. **p < 0.01, ***p < 0.001, as determined by Fisher's exact test. NS, Not significant. ***B***, Induction of *Psvh-2::nls::venus* expression in D-type motor neurons by laser surgery. Expression of fluorescent proteins in D-type motor neurons of wild-type, *sdz-33*, and *sdz-33*; *mdl-1* mutants 3 h after laser surgery is shown. White arrows indicate cell bodies of D-type neurons. Yellow arrowheads indicate the sites of laser surgery. D neurons are visualized by CFP under control of the *unc-25* promoter. Cell bodies of D-type neurons are magnified and shown within the red boxes. Most of the intestinal fluorescence in these photographs is from endogenous and variable background autofluorescence. A schematic representation of D-type motor neurons is shown in the right panel. Scale bars, 10 μm. ***C***, Quantification of the relative fluorescent levels of VENUS in D neurons with (+) or without (–) laser surgery (see Materials and Methods). The data are presented as box plot graph with median (thick line within the box) and interquartile range (edge of box). The numbers (*n*) of animals examined are shown. Statistical significance was determined using the Wilcoxon rank-sum test.

Our RNAi screen for *svh* genes was originally designed to identify components functioning in the JNK pathway (Li et al., 2012; Pastuhov et al., 2015). We next investigated where in this pathway SDZ-33 functions during axon regeneration. Activation of the JNK cascade following axonal injury is mediated by the SVH-2 Met-like RTK (Li et al., 2012). We confirmed that SDZ-33 functions in the same pathway as SVH-2 because the phenotype of *sdz-33(tm1210)*; *svh-2(tm737)* double mutants was indistinguishable from that of either single mutant (Fig. 3*A*, Table 3). We examined whether overexpression of *svh-2* might reverse the defect in axon regeneration observed in *sdz-33* mutants. We found that this was indeed the case (Fig. 3*A*, Table 3). These results suggest that SDZ-33 functions at or upstream of SVH-2 in the SVH-2–JNK pathway.

Since expression of *svh-2* is induced by axonal injury (Li et al., 2015), we examined whether SDZ-33 is involved in this induction using a reporter construct, *Psvh-2::nls::venus*, which consists of the *svh-2* promoter driving the fluorescent protein VENUS fused to NLS (Li et al., 2015). In wild-type animals, axon injury induced expression of *Psvh-2::nls::venus* in D-type motor neurons (Fig. 3*B*,*C*), as reported previously (Li et al., 2015; Sakai et al., 2019). However, in *sdz-33(tm1210)* mutants, no induction was observed (Fig. 3*B*,*C*). These results suggest that SDZ-33 is required for axotomy-induced *svh-2* expression.

### SDZ-33 targets MDL-1 for Ub-mediated degradation

**Figure 4.**
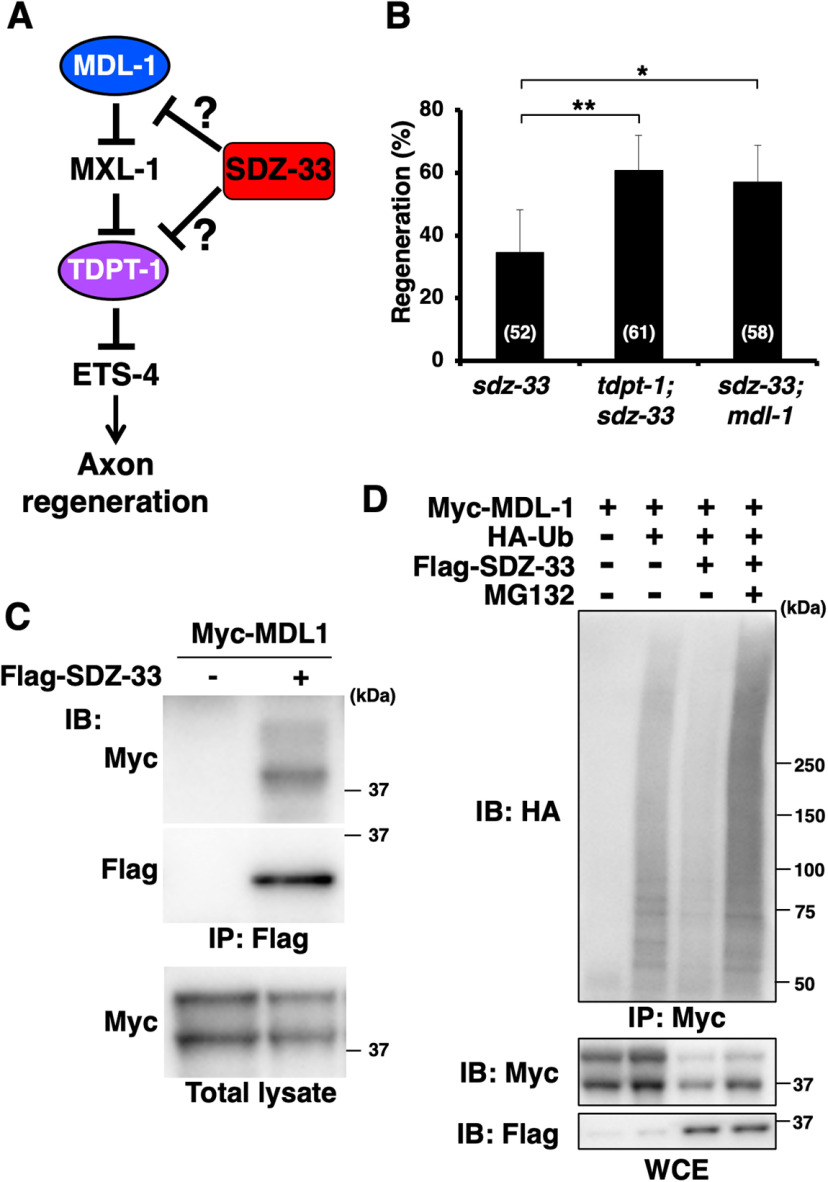
SDZ-33 targets MDL-1 for Ub-mediated degradation. ***A***, Inhibition of axon regeneration by MDL-1 and TDPT-1. ***B***, Percentages of axons that initiated regeneration 24 h after laser surgery. The numbers (*n*) of axons examined are shown. Error bars indicate 95% confidence intervals. *p < 0.05, **p < 0.01, as determined by Fisher's exact test. ***C***, Interaction of SDZ-33 with MDL-1. HEK293 cells were cotransfected with Myc-MDL-1 and Flag-SDZ-33, as indicated. Complex formation was detected by immunoprecipitation (IP) with anti-Flag antibody, followed by immunoblotting (IB) with the anti-Myc antibody. Total lysates were immunoblotted with anti-Myc antibody. ***D***, SDZ-33 mediates poly-ubiquitylation of MDL-1. COS-7 cells were transfected with Myc-MDL-1, HA-Ub, and Flag-SDZ-33, as indicated. Cells were incubated with or without MG132. Cell lysates were immunoprecipitated with anti-Myc antibody and immunoblotted with anti-HA antibody. Whole-cell extracts (WCEs) were analyzed by immunoblotting with anti-Myc and anti-Flag antibodies.

What is the target for SDZ-33 in the regulation of axon regeneration? F-box proteins are involved in protein degradation mediated by SCF E3-Ub ligase complexes (Craig and Tyers, 1999). Since SDZ-33 is required for axon regeneration, its target should be a negative regulator of axon regeneration. We recently demonstrated that axon injury-induced *svh-2* expression is negatively regulated by Mad-like MDL-1 and TDP2-like TDPT-1 (Fig. 4*A*; Sakai et al., 2019). We examined the effects of *mdl-1* and *tdpt-1* loss-of-function mutations on axon regeneration in *sdz-33* mutants. We found that both *mdl-1(tm311)* and *tdpt-1(km68)* mutations could suppress the defect in regeneration in *sdz-33(tm1210)* mutants (Fig. 4*B*, Table 3). As MDL-1 acts upstream of TDPT-1 in the axon regeneration pathway (Fig. 4*A*), these results suggest that SDZ-33 acts upstream of MDL-1 to induce *svh-2* expression in response to axon injury. Indeed, we confirmed that expression of *Psvh-2::nls::venus* in D-type neurons was induced by laser surgery in *sdz-33(tm1210)*; *mdl-1(tm311)* mutants (Fig. 3*B*,*C*).

Our genetic analysis of *mdl-1* and *sdz-33* above raised the possibility that SDZ-33 could act as a specific subunit in an SCF complex to mediate ubiquitylation of MDL-1, leading to its degradation. Since an F-box protein functions to target substrate proteins, we first examined whether SDZ-33 interacts with MDL-1 by cotransfecting mammalian HEK293 cells with Flag-tagged SDZ-33 and Myc-tagged MDL-1. Coimmunoprecipitation experiments revealed that Flag-SDZ-33 associated with Myc-MDL-1 (Fig. 4*C*). We next determined whether MDL-1 can be degraded by the SDZ-33-mediated 26S proteasome pathway in mammalian COS-7 cells. For this purpose, we coexpressed Myc-MDL-1 with HA-tagged Ub in COS-7 cells. Cell lysates were immunoprecipitated with anti-Myc antibody and immunoblotted with anti-HA antibody. We found that MDL-1 was poly-ubiquitylated (Fig. 4*D*), suggesting that endogenous mammalian E3-Ub ligases are capable of ubiquitylating MDL-1. We then asked whether SDZ-33 expression could cause ubiquitylation of MDL-1. COS-7 cells were cotransfected with Myc-MDL-1, HA-Ub, and Flag-SDZ-33. This assay assumes that *C. elegans* SDZ-33 interacts with the endogenous COS-7 SCF complex and provides substrate specificity for MDL-1. We found that coexpression of Flag-SDZ-33 decreased the levels of poly-ubiquitylated MDL-1 protein (Fig. 4*D*), suggesting that SDZ-33 promotes the degradation of ubiquitylated MDL-1. Consistent with this possibility, when cells were treated with MG132, a specific inhibitor of the 26S proteasome, the amounts of poly-ubiquitylated MDL-1 protein clearly increased (Fig. 4*D*). Thus, proteasome-mediated degradation contributes to the control of MDL-1 protein levels by SDZ-33. Together, the above results indicate that SDZ-33 is an F-box protein that directly targets MDL-1 for Ub-mediated degradation.

**Figure 5.**
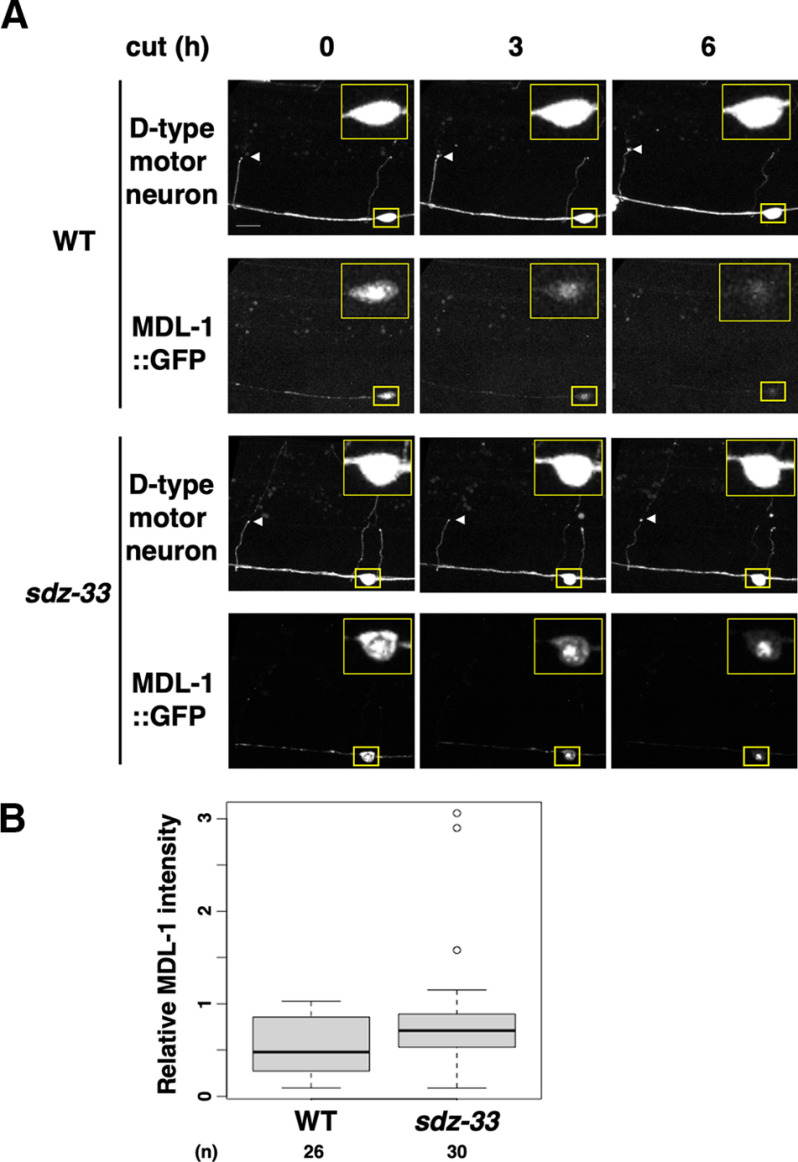
SDZ-33 promotes axotomy-induced nuclear degradation of MDL-1. ***A***, Fluorescent images of wild-type and *sdz-33* mutant expressing *Punc-47::mcherry* (D-type motor neuron, top) and *Punc-25:mdl-1::gfp* (bottom) are shown. Images were taken at 0, 3, or 6 h after laser surgery. White arrowheads indicate the tips of the severed axons. Cell bodies corresponding to the severed axons are outlined in yellow and are shown magnified in the insets. Scale bar, 10 µm. ***B***, Quantification of the fluorescent levels of MDL-1::GFP in the nucleus of D neurons. Relative MDL-1 intensity was calculated as a fraction of the relative MDL-1::GFP intensity 6 h after laser surgery divided by the corresponding value at 0 h postaxotomy (see Materials and Methods). The data are presented as a box plot graph with median (thick line within the box) and interquartile range (edge of box). White circles indicate outliers. The numbers (*n*) of axons examined are shown. Statistical significance was determined using the Wilcoxon rank-sum test; *p* = 0.0496.

We next investigated whether SDZ-33 regulates MDL-1 protein levels in animals expressing GFP-tagged MDL-1 from the *unc-25* promoter. As observed previously (Sakai et al., 2019), in wild-type animals MDL-1::GFP was found predominantly in the nucleus of D neurons, and its fluorescence intensity significantly decreased following axon injury (Fig. 5*A*,*B*). In contrast, we found that the *sdz-33(tm1210)* mutation resulted in significant stabilization of nuclear MDL-1::GFP levels, which persisted even 6 h after axotomy (Fig. 5*A*,*B*). These results provide evidence that SDZ-33 is involved in axon injury-induced degradation of MDL-1.

## Discussion

**Figure 6.**
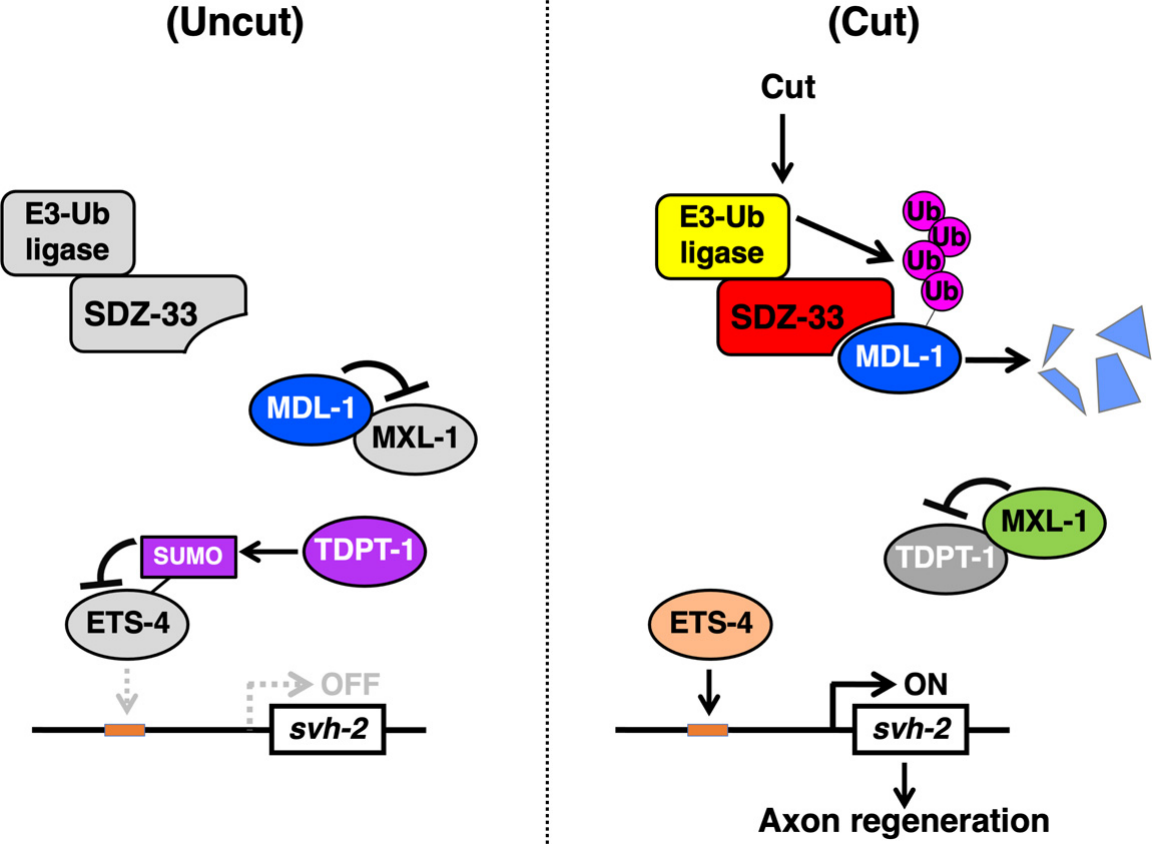
Schematic diagram of the role of SDZ-33 in axon regeneration. SDZ-33 recruits MDL-1 to the SCF E3-Ub ligase complex, leading to the subsequent destruction of MDL-1 by the 26S proteasome. MDL-1 degradation allows ETS-4 to activate *svh-2* expression.

In *C. elegans*, *svh-2* expression is upregulated in neurons following axon injury. This gene induction is mediated by a transcription factor complex composed of ETS-4 and CEBP-1 (Li et al., 2015). We have recently found that TDPT-1 inhibits axon injury-induced *svh-2* expression by inducing the SUMOylation of ETS-4, which inhibits formation of an ETS-4–CEBP-1 complex (Sakai et al., 2019). We have additionally identified a transcription factor, MXL-1, that acts as a positive regulator of *svh-2* expression in response to axon injury. However, in contrast to MXL-1, its partner transcription factor MDL-1 negatively regulates *svh-2* induction. Based on these findings, we propose the following molecular mechanism of how axon injury activates *svh-2* expression (Fig. 6). In the absence of axon damage, TDPT-1 maintains the SUMOylation of ETS-4, resulting in the repression of its transcriptional activity. Axon injury leads to the degradation of MDL-1, allowing free MXL-1 to interact with TDPT-1. This causes the release of ETS-4 from TDPT-1, resulting in ETS-4 deSUMOylation and consequent complex formation with CEBP-1. This ETS-4–CEBP-1 complex then induces *svh-2* expression.

By this model, there must be some mechanism by which axon injury induces the degradation of MDL-1. Here, we show that the *sdz-33* gene encoding an F-box protein is required for induction of *svh-2* gene expression following axon injury. F-box proteins serve as substrate recruitment factors for the SCF E3-Ub ligase (Craig and Tyers, 1999). SDZ-33 promotes poly-ubiquitylation of MDL-1, leading to its subsequent destruction by the 26S proteasome (Fig. 6). Deletion of *sdz-33* stabilizes MDL-1, which accumulates and is able to repress *svh-2* gene expression in response to axon injury. The ubiquitylation of target proteins is orchestrated by a Ub-activating enzyme (E1), a Ub-conjugating enzyme (E2), and an E3-Ub ligase, the latter of which is responsible for substrate recognition and Ub transfer. One E3-Ub ligase is the SCF complex, which is composed of Skp1, Cullin1, Rbx1, and an F-box protein (Craig and Tyers, 1999). Cullin functions as a scaffold that interacts with Skp1 and Rbx1 to form a complex generating the core E3-Ub ligase activity. F-box proteins selectively recruit target proteins and thereby mediate Ub interactions with their substrates. Thus, the F-box protein determines substrate specificity. F-box proteins contain an N-terminal F-box domain that binds to Skp1 to enable ubiquitylation of the target. F-box proteins often contain other motifs such as a WD40 repeat and a leucine-rich repeat, both of which are involved in substrate binding (Craig and Tyers, 1999). Identifying target substrates for F-box proteins would enhance our understanding of how SCF E3-Ub ligase mediates multiple biological functions. The *C. elegans* genome contains ∼700 genes encoding F-box proteins (Thomas, 2006), but only a few have been successfully assigned a function (Clifford et al., 2000; Kipreos et al., 2000; Liao et al., 2004; Fielenbach et al., 2007; Bounoutas et al., 2009; Wu et al., 2017). Here, we identify SDZ-33 as an F-box E3-Ub ligase that promotes the degradation of MDL-1. SDZ-33 lacks WD40 or leucine-rich repeats, but it does contain an FBA domain, which may be involved in the interaction with MDL-1. The *C. elegans* genome encodes >20 Skp1-related proteins and 6 Cullin proteins (Nayak et al., 2002). It would be interesting to determine which Skp1-related proteins or Cullin proteins are involved in SDZ-33-mediated degradation of MDL-1.

In the mammalian Myc/Mad/Max family of transcription factors, Myc forms a heterodimer with its partner Max. This Myc–Max complex then binds to the promoter region of target genes to activate their transcription. Mad1 suppresses Myc transcriptional activity by interacting with Max and inhibiting Myc–Max complex formation (Amati and Land, 1994). The *C. elegans mxl-1* and *mdl-1* genes encode homologs of vertebrate Max and Mad, respectively, but *C. elegans* lacks an obvious homolog of Myc (Pickett et al., 2007). MXL-1 has been shown to form a complex with MDL-1 and TDPT-1, and MDL-1 protein levels are tightly regulated in response to axon injury (Sakai et al., 2019). Therefore, the availability of MXL-1 to TDPT-1 SUMOylation is dependent on MDL-1 protein expression levels. The association of MXL-1 with TDPT-1 redirects the MXL-1–MDL-1 complex to regulate ETS-4. The results presented here suggest that Ub-mediated degradation regulates the Max–Mad network by promoting Mad degradation (Fig. 6). This work thus reveals an important module of this transcriptional regulatory system and illustrates the complex nature of the Max transcription factor network.

## References

[B1] Amati B, Land H (1994) Myc-Max-Mad: a transcription factor network controlling cell cycle progression, differentiation and death. Curr Opin Genet Dev 4:102–108. 10.1016/0959-437x(94)90098-1 8193530

[B2] Bounoutas A, Zheng Q, Nonet ML, Chalfie M (2009) mec-15 encodes an F-box protein required for touch receptor neuron mechanosensation, synapse formation and development. Genetics 183:607–617. 10.1534/genetics.109.105726 19652181 PMC2766320

[B3] Brenner S (1974) The genetics of Caenorhabditis elegans. Genetics 77:71–94. 4366476 10.1093/genetics/77.1.71 PMC1213120

[B4] Clifford R, Lee MH, Nayak S, Ohmachi M, Giorgini F, Shedl T (2000) FOG-2, a novel F-box containing protein, associates with GLD-1 RNA binding protein and directs male sex determination in the C. elegans hermaphrodite germline. Development 127:5265–5276. 11076749 10.1242/dev.127.24.5265

[B5] Craig KL, Tyers M (1999) The F-box: a new motif for ubiquitin dependent proteolysis in cell cycle regulation and signal transduction. Prog Biophys Mol Biol 72:299–328. 10.1016/s0079-6107(99)00010-3 10581972

[B6] Fielenbach N, Guardavaccaro D, Neubert K, Chan T, Li D, Feng Q, Hutter H, Pagano M, Antebi A (2007) DRE-1: an evolutionarily conserved F box protein that regulates C. elegans developmental age. Dev Cell 12:443–455. 10.1016/j.devcel.2007.01.018 17336909

[B7] Firnhaber C, Hammarlund M (2013) Neuron-specific RNAi in C. elegans and its use in a screen for essential genes required for GABA neuron function. PLoS Genet 9:e1003921. 10.1371/journal.pgen.1003921 24244189 PMC3820814

[B8] Hanafusa H, Ishikawa K, Kedashiro S, Saigo T, Iemura S-I, Natsume T, Komada M, Shibuya H, Nara A, Matsumoto K (2011) Leucine-rich repeat kinase LRRK1 regulates endosomal trafficking of the EGF receptor. Nat Commun 2:158. 10.1038/ncomms1161 21245839 PMC3105304

[B9] Hisamoto N, Matsumoto K (2017) Signal transduction cascades in axon regeneration: insights from C. elegans. Curr Opin Genet Dev 44:54–60. 10.1016/j.gde.2017.01.010 28213159

[B10] Hisamoto N, Li C, Yoshida M, Matsumoto K (2014) The C. elegans HGF/plasminogen-like protein SVH-1 has protease-dependent and -independent functions. Cell Rep 9:1628–1634. 10.1016/j.celrep.2014.10.056 25464847

[B11] Hisamoto N, Nagamori Y, Shimizu T, Pastuhov SI, Matsumoto K (2016) The C. elegans discoidin domain receptor DDR-2 modulates the Met-like RTK-JNK signaling pathway in axon regeneration. PLoS Genet 12:e1006475. 10.1371/journal.pgen.1006475 27984580 PMC5161311

[B12] Hisamoto N, Tsuge A, Pastuhov SI, Shimizu T, Hanafusa H, Matsumoto K (2018) Phosphatidylserine exposure mediated by ABC transporter activates the integrin signaling pathway promoting axon regeneration. Nat Commun 9:3099. 10.1038/s41467-018-05478-w 30082731 PMC6079064

[B13] Hisamoto N, Shimizu T, Asai K, Sakai Y, Pastuhov SI, Hanafusa H, Matsumoto K (2019) C. elegans Tensin promotes axon regeneration by linking the Met-like SVH-2 and integrin signaling pathways. J Neurosci 39:5662–5672. 10.1523/JNEUROSCI.2059-18.2019 31109965 PMC6636086

[B14] Kipreos ET, Gohel SP, Hedgecock EM (2000) The C. elegans F-box/WD-repeat protein LIN-23 functions to limit cell division during development. Development 127:5071–5082. 11060233 10.1242/dev.127.23.5071

[B15] Li C, Hisamoto N, Nix P, Kanao S, Mizuno T, Bastiani M, Matsumoto K (2012) The growth factor SVH-1 regulates axon regeneration in C. elegans via the JNK MAPK cascade. Nat Neurosci 15:551–557. 10.1038/nn.3052 22388962

[B16] Li C, Hisamoto N, Matsumoto K (2015) Axon regeneration Is regulated by Ets-C/EBP transcription complexes generated by activation of the cAMP/Ca^2+^ signaling pathways. PLoS Genet 11:e1005603. 10.1371/journal.pgen.1005603 26484536 PMC4618690

[B17] Liao EH, Hung W, Abrams B, Zhen M (2004) An SCF-like ubiquitin ligase complex that controls presynaptic differentiation. Nature 430:345–350. 10.1038/nature02647 15208641

[B18] Mello CC, Kramer JM, Stinchcomb D, Ambros V (1991) Efficient gene transfer in C. elegans: extrachromosomal maintenance and integration of transforming sequences. EMBO J 10:3959–3970. 10.1002/j.1460-2075.1991.tb04966.x 1935914 PMC453137

[B19] Mizuno T, Hisamoto N, Terada T, Kondo T, Adachi M, Nishida E, Kim DH, Ausubel FM, Matsumoto K (2004) The Caenorhabditis elegans MAPK phosphatase VHP-1 mediates a novel JNK-like signaling pathway in stress response. EMBO J 23:2226–2234. 10.1038/sj.emboj.7600226 15116070 PMC419906

[B20] Nayak S, Santiago FE, Jin H, Lin D, Schedl T, Kipreos ET (2002) The Caenorhabditis elegans Skp1-related gene family: diverse functions in cell proliferation, morphogenesis, and meiosis. Curr Biol 12:277–287. 10.1016/s0960-9822(02)00682-6 11864567

[B21] Nix P, Hisamoto N, Matsumoto K, Bastiani MJ (2011) Axon regeneration requires co-activation of p38 and JNK MAPK pathways. Proc Natl Acad Sci U S A 108:10738–10743. 10.1073/pnas.1104830108 21670305 PMC3127873

[B22] Pastuhov SI, Fujiki K, Nix P, Kanao S, Bastiani M, Matsumoto K, Hisamoto N (2012) Endocannabinoid-Goα signalling inhibits axon regeneration in Caenorhabditis elegans by antagonizing Gqα-PKC-JNK signalling. Nat Commun 3:1136. 10.1038/ncomms2136 23072806 PMC3493645

[B23] Pastuhov SI, Hisamoto N, Matsumoto K (2015) MAP kinase cascades regulating axon regeneration in C. elegans. Proc Jpn Acad Ser B Phys Biol Sci 91:63–75. 10.2183/pjab.91.63 25792136 PMC4410086

[B24] Pickett CL, Breen KT, Ayer DE (2007) A C. elegans Myc-like network cooperates with semaphorin and Wnt signaling pathways to control cell migration. Dev Biol 310:226–239. 10.1016/j.ydbio.2007.07.034 17826759 PMC2077855

[B25] Robertson SM, Shetty P, Lin R (2004) Identification of lineage-specific zygotic transcripts in early Caenorhabditis elegans embryos. Dev Biol 276:493–507. 10.1016/j.ydbio.2004.09.015 15581881

[B26] Sakai Y, Hanafusa H, Pastuhov SI, Shimizu T, Li C, Hisamoto N, Matsumoto K (2019) TDP2 negatively regulates axon regeneration by inducing SUMOylation of an Ets transcription factor. EMBO Rep 20:e47517. 10.15252/embr.201847517 31393064 PMC6776894

[B27] Shimizu T, Pastuhov SI, Hanafusa H, Matsumoto K, Hisamoto N (2018) The C. elegans BRCA2-ALP/Enigma complex regulates axon regeneration via a Rho GTPase-ROCK-MLC phosphorylation pathway. Cell Rep 24:1880–1889. 10.1016/j.celrep.2018.07.049 30110643

[B28] Shimizu T, Kato Y, Sakai Y, Hisamoto N, Matsumoto K (2019) N-glycosylation of the discoidin domain receptor is required for axon regeneration in Caenorhabditis elegans. Genetics 213:491–500. 10.1534/genetics.119.302492 31371405 PMC6781908

[B29] Tedeschi A, Bradke F (2017) Spatial and temporal arrangement of neuronal intrinsic and extrinsic mechanisms controlling axon regeneration. Curr Opin Neurobiol 42:118–127. 10.1016/j.conb.2016.12.005 28039763

[B30] Thomas JH (2006) Adaptive evolution in two large families of ubiquitin-ligase adapters in nematodes and plants. Genome Res 16:1017–1030. 10.1101/gr.5089806 16825662 PMC1524861

[B31] Wu CW, Wang Y, Choe KP (2017) F-box protein XREP-4 is a new regulator of the oxidative stress response in Caenorhabditis elegans. Genetics 206:859–861. 10.1534/genetics.117.200592 28341649 PMC5499191

[B32] Yan D, Wu Z, Chisholm AD, Jin Y (2009) The DLK-1 kinase promotes mRNA stability and local translation in C. elegans synapses and axon regeneration. Cell 138:1005–1018. 10.1016/j.cell.2009.06.023 19737525 PMC2772821

[B33] Yanik MF, Cinar H, Cinar HN, Chisholm AD, Jin Y, Ben-Yakar A (2004) Neurosurgery: functional regeneration after laser axotomy. Nature 432:822. 10.1038/432822a 15602545

